# Coverage, universal access and equity in health: a characterization of scientific production in nursing

**DOI:** 10.1590/1518-8345.1082.2669

**Published:** 2016-03-04

**Authors:** Sara Mendoza-Parra

**Affiliations:** 1 PhD, Professor, Facultad de Enfermería, Universidad de Concepción, Concepción, Chile

**Keywords:** Universal Coverage, Universal Access to Health Care Services, Equity in Health, Bibliometrics, Nursing

## Abstract

**Objectives:**

to characterize the scientific contribution nursing has made regarding coverage, universal access and equity in health, and to understand this production in terms of subjects and objects of study.

**Material and methods:**

this was cross-sectional, documentary research; the units of analysis were 97 journals and 410 documents, retrieved from the Web of Science in the category, "nursing". Descriptors associated to coverage, access and equity in health, and the Mesh thesaurus, were applied. We used bibliometric laws and indicators, and analyzed the most important articles according to amount of citations and collaboration.

**Results:**

the document retrieval allowed for 25 years of observation of production, an institutional and an international collaboration of 31% and 7%, respectively. The mean number of coauthors per article was 3.5, with a transience rate of 93%. The visibility index was 67.7%, and 24.6% of production was concentrated in four core journals. A review from the nursing category with 286 citations, and a Brazilian author who was the most productive, are issues worth highlighting.

**Conclusions:**

the nursing collective should strengthen future research on the subject, defining lines and sub-lines of research, increasing internationalization and building it with the joint participation of the academy and nursing community.

## Introduction

Under the assumption that universal health coverage cannot be achieved without scientific data provided by research - therefore, research becomes an undeniable instrument to solve the diversity of questions about how to achieve the universality of care health^(^ 1 ^)^ - the present investigation formulated the following question: What are the characteristics of the nursing contribution in terms of scientific production, subjects and events of interest, regarding coverage, access and universal health equity? Two conceptual references were used to answer this question: universal health coverage and bibliometrics. 

Universal coverage or universal health coverage consists of the development of health financing systems that enable all people to have access to health services, including advocacy, prevention, treatment and rehabilitation activities, and that their having access incurs no financial difficulties for them in terms of paying for those services. In other words, it involves solving how the health system is financed, how it protects people from the financial consequences that facing an illness brings to them for the needed care, as well as how the resources available in that system are optimally used^(^ 2 ^-^ 3 ^)^. Thus two other essential concepts arise: access and equity. Universal access in health means an absence of geographic, economic, sociocultural, and organizational or gender barriers, and this is achieved through the progressive removal of barriers that prevent people from using all comprehensive health services, determined equitably and at a national level^(^ 4 ^)^. In turn, universal health equity is a broad, inclusive, and multidimensional concept, consisting of aspects related to achieving good health through processes that not only have to do with the distribution of health care, but also with social justice and non-discrimination in the delivery of such care^(^ 5 ^)^. Health is not only a function of the health sector; in order to achieve it, other factors such as living conditions and working conditions, psychosocial factors and socioeconomic status are involved^(^ 6 ^)^. In other words, universal equity in health means achieving health without any social circumstance to prevent it^(5).^

Once the conceptual contribution of the study was determined, a bibliometric analysis was used for characterization because, while it allows us to look back at how scientific advances have been achieved and released, it also reveals the generation of useful results and measures the development of scientific disciplines on certain research lines^(^ 7 ^)^. Bibliometric indicators of production quantify both the number of documents published, by country, institution and authors, as well as the citations of those documents as a measure of their impact or importance^(^ 8 ^)^. These are measurements obtained from the statistical analysis of the basic elements of journals or articles with which indicators are built to measure the quality, impact, relationships or collaboration and scientific activity, i.e., the quantification and temporal evolution of the production^(^ 9 ^)^ . Thus, the information stored in databases represents raw material which, once analyzed, allows for the extraction of knowledge that can contribute to understanding scientific efforts and making strategic decisions in a particular field of knowledge^(^ 10 ^)^. 

Based on the information above, this study had two objectives: first, to characterize the scientific contribution of nursing in coverage, universal access and equity in health through bibliometric indicators; and, second, to understand the trends of this production in terms of studied subjects and phenomena .

## Materials and methods

The type of study and unit of analyze was: bibliometric, descriptive and exploratory research, whose unit of analysis was serial publications and documents recovered from the category, "Nursing", in the Web of Science, henceforth WOS. 

**Figure 1:**
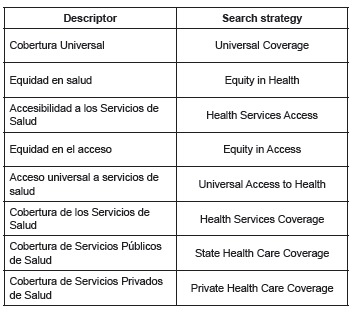
- Descriptors and search strategies, Nursing Category, Web of Science, 2015

Search strategies (Figure 1) were: a consultation of DeCS (Descriptors in Health Sciences) and MeSH (Medical Subject Heading) thesaurus was conducted to recover those controlled terms or descriptors that were related to the natural terms of the research problem, i.e. "coverage", "access" and "equity", and each separately associated with the terms "health", "health care", "health services" and "universal". 

The universe consisted of: 97 magazines and 410 documents (articles, reviews, letters, notes, editorials, etc.). Data analysis used was: frequency distribution and measures of central tendency and dispersion for exploratory univariate analysis of unidimensional or descriptive bibliometric indicators. For the behavior of authors and journals, the laws of Lotka and Bradford were calculated.

## Results

### Document characteristics

**Figure 2:**
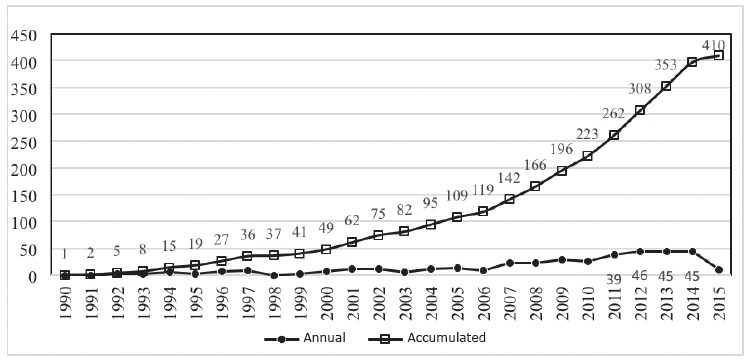
- Scientific production of nurses in subjects on Coverage, Access and Universal Equity in health, Web of Science, 1990-2015 (N = 410).

Magnitude, evolution and document types (Figure 2): registration of documents in the WOS starting in the 1990s, with an article, reaching an average of 47.5, from 2011 on. By 2015, there were already a total of 410 records. According to the type of documents, in the 25 years studied, 91.5% (375) were articles, 3.7% (15) were reviews and 2.2% (9) were editorials. The rest were brief reports, news, and letters to the editor. 

Collaboration: only 31% of this production (127) had institutional collaboration; the most common partnership (27) was university/hospital. The rate of collaboration or co-authorship, i.e., the average number of authors per paper, throughout the period was 3.5. The highest value was obtained by a co-authored article from 2007,^(^ 11 ^)^ with 12 Canadian authors, which described how the real-time access that hospital or community nurses had to electronic resources could not only obtain information simultaneously about patient results or about the best evidence to support the practice, but also revealed the close relationship between nursing interventions and patient outcomes. This is a key issue in planning the best interventions in a timely manner, and thereby facilitates access to better health care in the short term^(^ 11 ^)^. Collaboration at a national level exceeded this indicator in 93.4% of the manuscripts (383), of which, the document with the highest number of authors' participating affiliations was from 2009, in which nine academic Australian philanthropic, research and care institutions were identified; their purpose was to conduct a mapping of rural and regional cancer services in that country. Among other things, significant deficiencies in the provision of oncological services, limited availability of nurses, and significant differences in their training to provide similar care were identified. It was concluded that these deficiencies could contribute to disadvantage the progress of cancer patients living in regional, rural and remote areas, and that it was imperative to take short-term measures to improve access to better oncological services and thereby eliminate inequalities in the care of these patients in Australia^(^ 12 ^)^. International collaboration only reached 7%, led by the United States, with ten publications along with European, Asian, African and Latin American countries. Notable in this group was a manuscript, with the increased presence of participating countries, with authors from England, Norway, Australia, Switzerland, Germany and Greece. It was a review published in 2013, from 16 studies, describing the perceptions, needs and experiences of pregnant migrant women. It was concluded that although all member states of the European Union had ratified resolutions based on human rights, a connection remained between social inequality and barriers to accessing pre-, intra- and post-partum care. The results showed that migrant women were a very vulnerable group during pregnancy and childbirth, and it was necessary to improve access to health services to better meet their actual needs^(^ 13 ^)^. 

Authors, occasional production and Lotka law: 1197 authors were identified in all of the papers. Of these, 1109 had only one article, so the occasional production or transience rate was 93%. Because of this ratio, we could not get a predictable standard distribution of authors and studies. In accordance with the foregoing, by calculating the Lotka coefficient (-3.4), it was found that the community of authors in regards to coverage, universal access and health equity in the WOS Nursing category remains a community of researchers with poor scientific productivity, represented by an elite group of 34 researchers (√1197) who have written three or more papers. Herein, the author Tereza Cristina Scatena Villa stands out, who heads the elite as a co-author of nine articles published from 2011 to 2014, which cover various topics regarding accessibility to tuberculosis care services.

Quotations: visibility index, i.e., the relative weight that had the number of citable documents (articles, reviews, letters and notes), in this case 394, which were cited during the period, was 67.7% (267). It seemed important to investigate the behavior of scientific citation by scientific category, given that 118 of the citable documents (29.9%) were linked to one or more of the 18 categories found, other than "Nursing". Table 1 shows the average citation per category. The highest average number of citation (7.7) reached up to six documents, related to the Obstetrics & Gynecology categories, followed by two related to Public, Environment & Occupational Health and thirdly, those linked to Cardiology & Cardiovascular System. The Nursing category, in the 25 years studied, ranked fourth with an average of six citations for 394 documents. 

**Table 1:** - scientific categories according to the average number of citations and documents, Nursing Category, Web of Science, 2015

**Scientific category**	**Quote X**	**Number of quotable documents**
Obstetrics & Gynecology	7.7	6
Public, Environmental & Occupational Health	7.5	51
Cardiovascular System & Cardiology	6.8	5
Nursing	6	394
Oncology	5	15
Pediatrics	4.7	9
Health Care Sciences & Services	4.5	13
Psychiatry	4	5
Medical Informatics	3.6	5
Computer Science	3.6	5
Gastroenterology & Hepatology	3	1
Rehabilitation	2.9	8
Education & Educational Research	2.5	2
Geriatrics & Gerontology	1.2	5
Substance Abuse	1	3
Emergency Medicine	0	2
Urology & Nephrology	0	1
Criminology & Penology	0	1
Life Sciences & Biomedicine - Other Topics	0	1
Integrative & Complementary Medicine	0	1

### Journal characteristics

Country of Origin: Table 2 identifies 97 journals originating from 15 countries. Four countries stand out with the largest number of source journals on the subject: USA, England, Brazil and Australia. 

**Table 2:** - Distribution of countries of source journals by number of journals and documents, Nursing Category, Web of Science, 2015

**Country**	**Number of Journals**	**Number of Documents**
USA	55	207
UK	13	73
Brazil	7	72
Australia	5	33
Switzerland	2	6
Spain	3	4
Japan	1	3
Korea	3	3
Scotland	1	2
Portugal	2	2
Colombia	1	1
Cuba	1	1
The Netherlands	1	1
South Africa	1	1
Taiwan	1	1
Total	97	410

Core journals (Bradford Law): based on the number of documents per journal, four Bradford areas were identified (Table 3), each with a production rate ranging between 23% and 26%. There were five core journals, i.e., those in which the largest scientific production regarding coverage, universal access and equity in health was concentrated: Public Health Nursing from the USA, with 32 documents; Journal of Advanced Nursing from the UK, with 23 documents; Australian Journal of Rural Health with 17 documents; and two Brazilian journals, *Revista da Escola de Enfermagem da USP* and *Acta Paulista de Enfermagem*, with 15 and 14 articles, respectively.

**Table 3:** - Areas of Bradford, concerning coverage, universal access and equity in health, Category Nursing, Web of Science, 2015

**Number ofjournals**	**Number of articles per journal**	**Accumulated journals, r**	**Accumulated articles, R ( r )**	**Bradford Areas**
1	32	1	32	Zone 1 Nucleus 5 journals (24.6%)
1	23	2	55	
1	15	3	70	
1	17	4	87	
1	14	5	101	
2	13	7	127	Zone 2 9 journals (23.2%)
1	12	8	139	
1	11	9	150	
1	10	10	160	
4	9	14	196	
3	8	17	220	Zone 3 19 journals (26.3%)
3	7	20	241	
3	6	23	259	
5	5	28	284	
5	4	33	304	
13	3	46	343	Zone 4 64 journals (25.9%)
16	2	62	375	
35	1	97	410	

### Study subjects and phenomena

**Figure 3:**
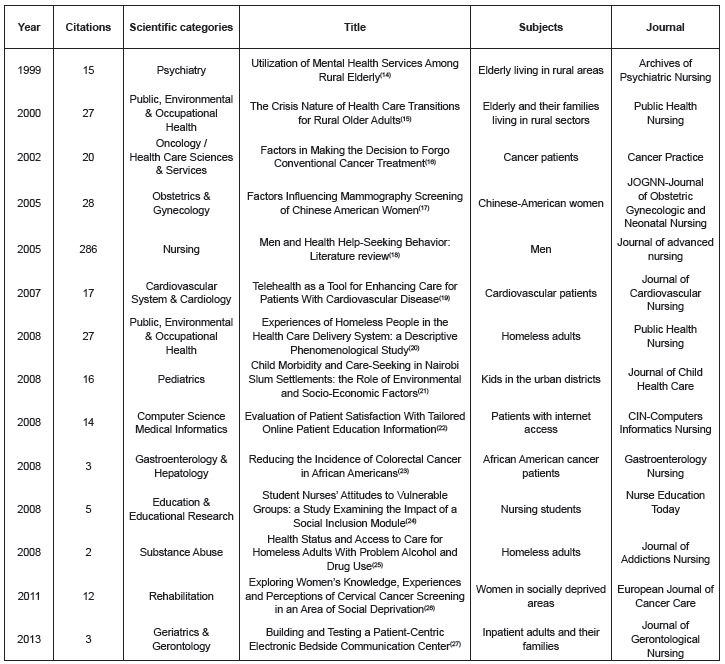
- Summary of documents related to coverage, universal access and equity in health, by years, citations, subject and journal, Nursing Category, Web of Science, 2015

To monitor trends on topics of interest, it seemed important to retrieve and analyze, by years, the most cited documents separated by scientific categories, and separate from them the phenomena and study subjects (Figure 3). A total of 15 categories in the 394 scientific papers, and 14 citable documents with the highest number of citations by category were identified. In the most cited group, a 2005 review with 286 citations supporting the Nursing category is highlighted.

In the UK, based on growing American evidence that men seek help to solve several health problems, such as depression, substance abuse, physical disabilities or stressful life events less frequently than women and that men were more reluctant to seek help and access health services, there was a need to retrieve scientific literature to describe such behavior. A growing number of gender studies were found that showed a tendency for men to delay seeking help when they got sick. Under this approach, white middle-class men, for example, had a*traditional male behavior* that prevented them from accessing timely health care. It was concluded that, primarily, the role of male beliefs and the similarities and differences between different male profiles required more attention and research; especially taking into account health inequalities that existed between men of different socio-economic status and ethnicity. The authors suggested further research with heterogeneous samples to obtain a better understanding of the triggers and barriers associated with the process of making decisions about men seeking care health. They emphasized that, with more evidence, not only could it improve the access of men to health care, but it would also improve the quality of life of the women who informally have to take over that care, and therefore also reduce national health costs associated with delayed health care^(^ 18 ^)^. 

## Discussion and conclusions

It is worth highlighting that the entry of Scielo and KCI-Korean collections in the Web of Science enables the performance of global bibliometric studies, in order to observe the behavior of particular scientific fields and study phenomena. While it is arguable that this study does not include everything the nursing community has produced regarding coverage, access and universal equity in health, from WOS, we can separate the collection of documents with the best scientific quality about the subject, and have an overview of their contribution. 

With respect to documents, specifically regarding the evolution of magnitude, it is clearly shown that the issue has not entered the phase of linear growth. This is a characteristic that lends to investigative work with a scientific maturity of an area of knowledge, even if the 25-year period studied was enough to strengthen this line of research. Evidence that reinforces this includes the small amount of retrieved reviews (3.7%) and primarily national collaboration (93.4%) which originated from the university environment with high levels of transience. This coincides with the point made by Trzesniak^(^ 28 ^)^ to establish that nursing, as with other emerging sciences, is still in transition from technology/profession into science/research, and the behavior of the group that has researched coverage, access and equity in health, proves it. 

It is remarkable that, despite the increased productivity originating in countries such as the USA and UK, it is a Brazilian author that leads the productivity rates by author. Indeed, Scatena Villa has had a continuing involvement over time with nine articles that may well represent a research subline regarding coverage, access and equity in patients with tuberculosis.

The visibility achieved by the citable documents (67%) is also important, and almost 30% are categorized in other scientific fields, indicating that those papers produced by Nursing on the issue not only impact its own group, but also contribute to the knowledge of other scientific areas^(^ 9 ^)^ .

Regarding the journals, it is expected that countries with a lot of data in the WOS occupy the greatest amount of indexed journals, which makes it more relevant that Brazil is integrated into the group of core journals, and ranks third with seven journals which accumulate 72 full text documents with free access. No doubt, this free access incorporated into a global database such as the WOS could be called*global scientific accessibility*. This is a key and important feature in order to ethically meet universal health coverage. Without access to relevant science produced around the world, one cannot speak of universality or coverage or access, and, even less, of equity in health. 

Regarding the featured articles, either in collaboration or citations, the set of documents indicates that nursing has understood very well the concepts of coverage, access and universal equity in health. The fact that the study subjects are elderly in rural areas, cancer patients, patients with psychiatric illnesses, pregnant migrant women, homeless adults, children living in slums, adult men hospitalized or unwilling to access health care, and many other types of groups, reflects an understanding of the need to progressively remove all barriers that prevent people from using the comprehensive health services, organized by the countries to which they belong^(^ 29 ^)^. Likewise, the fact that the research phenomena most frequently cited related to decision-making in health, use of health services, factors affecting diagnostics, mean usage, user satisfaction, attitudes of nursing students confronting vulnerable groups, or use of computer networks and tools to educate or connect the family with the inpatient, indicate that nursing seeks, through its research, those niches of inequality and inequity in care as a mechanism to clearly observe reality and reduce those gaps that impede universality in health care. 

Although neither the methodological framework nor the theoretical research in the study were part of this investigation, we observed a variety of qualitative and quantitative approaches to solve research problems, and the gender perspective as a fundamental perspective to build knowledge in this area. Gender and universal coverage, access and equity in health care perspectives have their fundamental livelihood in human rights and ethics.

It is a fact that the worldwide nursing community already has core journals in the area, but there remains a need to strengthen future research by better organizing it in terms of research lines and sub lines, internationalization, and creating a close link between academia, now linked to nursing, which is developed at a community level.
